# Behaviours and attitudes in response to the COVID-19 pandemic: insights from a cross-national Facebook survey

**DOI:** 10.1140/epjds/s13688-021-00270-1

**Published:** 2021-04-14

**Authors:** Daniela Perrotta, André Grow, Francesco Rampazzo, Jorge Cimentada, Emanuele Del Fava, Sofia Gil-Clavel, Emilio Zagheni

**Affiliations:** 1 Max Planck Institute for Demographic Research, Konrad-Zuse-Straße 1, Rostock, Germany; 2 Saïd Business School, Leverhulme Centre for Demographic Science, and Nuffield College, University of Oxford, Park End St., Oxford, United Kingdom

**Keywords:** COVID-19, Facebook surveys, Public health policy, Human behaviour

## Abstract

**Background:**

In the absence of medical treatment and vaccination, individual behaviours are key to curbing the spread of COVID-19. Here we describe efforts to collect attitudinal and behavioural data and disseminate insights to increase situational awareness and inform interventions.

**Methods:**

We developed a rapid data collection and monitoring system based on a cross-national online survey, the “COVID-19 Health Behavior Survey”. Respondent recruitment occurred via targeted Facebook advertisements in Belgium, France, Germany, Italy, the Netherlands, Spain, the United Kingdom, and the United States. We investigated how the threat perceptions of COVID-19, the confidence in the preparedness of organisations to deal with the pandemic, and the adoption of preventive and social distancing behaviours are associated with respondents’ demographic characteristics.

**Results:**

We analysed 71,612 questionnaires collected between March 13-April 19, 2020. We found substantial spatio-temporal heterogeneity across countries at different stages of the pandemic and with different control strategies in place. Respondents rapidly adopted the use of face masks when they were not yet mandatory. We observed a clear pattern in threat perceptions, sharply increasing from a personal level to national and global levels. Although personal threat perceptions were comparatively low, all respondents significantly increased hand hygiene. We found gender-specific patterns: women showed higher threat perceptions, lower confidence in the healthcare system, and were more likely to adopt preventive behaviours. Finally, we also found that older people perceived higher threat to themselves, while all respondents were strongly concerned about their family.

**Conclusions:**

Rapid population surveys conducted via Facebook allow us to monitor behavioural changes, adoption of protective measures, and compliance with recommended practices. As the pandemic progresses and new waves of infections are a threatening reality, timely insights from behavioural and attitudinal data are crucial to guide the decision-making process.

**Supplementary Information:**

The online version contains supplementary material available at 10.1140/epjds/s13688-021-00270-1.

## Background

As of mid-April 2020, the COVID-19 pandemic had already caused over 1.9 million cases and over 120,000 deaths worldwide [1]. Government responses have varied considerably across countries, applying a variety of non-pharmaceutical interventions to reduce movements and contacts in the population to mitigate the burden of COVID-19. Achieving universal adoption of public health recommendations is critical to curbing the spread of COVID-19 worldwide, especially when effective medical treatment and vaccination are not available [2]. However, in Western democracies, individual behaviours, rather than governmental actions, are potentially crucial for controlling the spread of COVID-19 in the long run [3]. Human behaviour is indeed a key factor in shaping the course of epidemics, as changes in behaviours translate into changes in the epidemic itself, directly affecting the likelihood of transmission and infection [4–6]. Understanding how the members of different demographic groups perceive the risk, and consequently adopt preventive behaviours, is therefore crucial to increase situational awareness and inform interventions.

In this context, we have developed and launched a rapid data collection and monitoring system based on a cross-national online survey, called the “COVID-19 Health Behavior Survey” (CHBS). Our goal was to gather key insights on people’s behavioural responses to the pandemic in multiple countries. We used a novel approach to recruit respondents via Facebook advertising campaigns, which allowed us to quickly engage a large number of Facebook users to fill in our questionnaire in a timely, cost-effective, and comparative manner. To correct for non-representativeness, we apply a post-stratification weighting approach commonly employed in survey research to approximate a representative sample of the population in each country [7–9].

In this paper, we investigate how different demographic groups of respondents differ in (i) their perception of the threat posed by COVID-19, (ii) their confidence in the preparedness of various organisations to handle the pandemic, and (iii) the uptake of preventive and social distancing behaviours. Our analysis is based on 71,612 completed questionnaires collected from March 13 to April 19, 2020 in Belgium, France, Germany, Italy, the Netherlands, Spain, the United Kingdom, and the United States. From a public health perspective, our findings provide key insights into behavioural changes and people’s compliance with recommended measures, which is relevant for policy makers in designing adequate communication campaigns and control strategies, especially in the current circumstances of new waves of infections. Note that given the relevance of the topic, a first draft of this manuscript has been posted on *medRxiv* to allow the scientific community to access our results [10].

## Methods

### Study design

Our questionnaire had four sections: (i) socio-demographic indicators; (ii) health indicators; (iii) attitudes and behaviours in response to COVID-19; (iv) social contacts. To facilitate validation and comparability with existing surveys, we included questions from several sources, such as the European Social Survey (ESS) [11], Ipsos [12], and the Polymod study [13]. We created the questionnaire first in English and then translated it with support from professional translators, considering country-specific differences, where applicable (e.g., differences in the educational system). The online version was implemented in LimeSurvey and was available in both English and the national or primary language(s) of each country. The full English questionnaire (as used in the United States) is reported in Additional file 2.

To recruit participants for our survey, the link to the questionnaire was distributed through Facebook advertising campaigns that we created via the Facebook Ads Manager (FAM). The FAM enables advertisers to create advertising campaigns that can be targeted at specific user groups, as defined by their demographic characteristics (e.g., sex and age) and a set of characteristics that Facebook infers from their behaviour on the network (e.g., interests). Following the methodology in Pötzschke and Braun [14], we opted for targeted advertising campaigns to disseminate our survey homogeneously across different demographic groups. Specifically, we created one advertising campaign per country and stratified each campaign by sex (male and female), age (18-24, 25-44, 45-64, and 65+ years), and region of residence (see the complete list of regions in Supplementary Table S4, Additional file 1). Note that Facebook users were shown the ads in only one language, while in the questionnaire itself they could choose between English and the national or primary language(s) of the country. This approach resulted in 24 to 56 strata per country, further stratified using six different ad images to ensure interest to a wide audience. Figure S1, Additional file 1, illustrates the structure of the advertising campaign used in the United States. More details about the recruitment strategy in the study can be found in Ref. [15].

We launched the campaigns on March 13, 2020, in Italy, the United Kingdom, and the United States. We then added Germany and France on March 17, Spain on March 19, the Netherlands on April 1, and Belgium on April 4, 2020. The countries included in this study were selected based on the following factors: (i) the initial size of the COVID-19 burden when we launched the survey; (ii) the availability of previously collected data to facilitate validation and comparison (e.g., the Polymod study on social contacts [13] and participatory surveillance systems like Influenzanet [16]); (iii) country-level expertise in our research team.

### Data pre-processing

We select respondents who reported their sex, age, and region of residence, and who were aware of the COVID-19 outbreak (Q12 of the questionnaire) and were therefore asked further questions about it (Sect. 4 of the questionnaire). As shown in Supplementary Table S2, Additional file 1, the latter filter excludes less than 1% of respondents. We excluded the data collected in Spain in week 2020-12 due to the small number of completed questionnaires (<100).

After respondent selection, we apply a post-stratification weighting approach in order to correct for potential issues with non-representativeness in the sample. This is a standard procedure in survey research, in which appropriate weights are computed based on population information from representative data sources (e.g., census data). Here, we use population data from Eurostat (2019) [17] and the US census (2018) [18]. The weights $w_{i}$ are defined as the ratio between the proportion $p_{i}$ of the true population and the proportion $\hat{p}_{i}$ of the sample population in each stratum *i* (i.e. combination of sex, age, and macro-region). More details on the post-stratification approach can be found in Supplementary Sect. 3, Additional file 1.

### Data analysis

We focus on three variables: (i) threat perceptions of COVID-19, (ii) confidence in various organisations to deal with the pandemic, and (iii) uptake of preventive and social distancing measures. Note that in the questionnaire we asked respondents to report their sex (male/female), which may or may not align with their gender identity. For simplicity, we use the terms male/female and man/woman interchangeably from here on, without any implication that biological sex and gender are exactly the same, or that differences in behaviours and attitudes between men and women that we report here are biologically determined.

We asked respondents to rate the threat that COVID-19 poses to themselves, their family, their local community, their country, and the world (Q13), on a 5-point Likert-type scale (1 = very low threat, 5 = very high threat). As a reference, we asked the same questions also for influenza (Q30). Furthermore, we asked respondents to rate their confidence in the preparedness of various organisations to effectively deal with the COVID-19 pandemic (Q14) on a 4-point Likert-type scale (1 = not confident at all, 4 = very confident). Here we consider five items, namely the confidence in the local healthcare system (as the average confidence in doctors/healthcare professionals and in local hospitals), the national healthcare system, the World Health Organization (WHO), the local government, and the national government. In Additional file 1, we report descriptive plots showing the distributions of the perceived threat posed by COVID-19 and influenza (Supplementary Figures S4 and S5), and the distributions of the confidence in organisations (Supplementary Figures S6 and S7). Note that all distributions are unimodal, except for the confidence in the national government in Spain and the United States, which show slight deviations from unimodality.

To create scores of perceived threat and confidence, we normalized respondents’ answers to each item to the range 0-1, corresponding to low and high perceived threat/confidence, respectively. The options “Don’t know” and “Prefer not to answer” are treated as missing values (see Supplementary Table S3, Additional file 1, reporting the sample size for each item).

We also asked respondents to report which measures, if any, they had taken to protect themselves from COVID-19 (Q18). This question was largely inspired by an Ipsos survey [12] and consists of a list of actions from which respondents could choose all that apply. It is therefore treated as dichotomous and interpreted as “Yes” if the respondents checked the item on the list or “No” if left blank, determining whether or not the corresponding behaviour was adopted with respect to the pre-pandemic period. We compute adoption rates as the weighted proportion of respondents who reported a specific behaviour, including: (i) stockpiling food and/or medicine; (ii) wearing a face mask; (iii) more frequent use of hand sanitizer; (iv) more frequent hand washing; (v) increased social distancing (if respondents selected at least one of the following: avoided shaking hands, avoided social activities, or avoided crowded places); (vi) reduced use of public transportation (if respondents selected at least one of the following: avoided travelling by public transportation, or avoided travelling by taxi). Note that while for most of the measures listed in Q18 the adoption rate is straightforward for capturing changes in behaviour (e.g., washing hands more often than before), changes in other behaviours are more difficult to assess due to the lack of a pre-pandemic baseline (e.g., not travelling by taxi or public transport even before the pandemic).

We used non-parametric tests for median comparisons (Wilcoxon test to compare two groups and Kruskall–Wallis test to compare three or more groups) and considered *p*-values of less than 0.05 to be significant. Data analysis was performed with Python (version 3.7) using the following packages and libraries: *pandas* (1.2.1) for data manipulation, *scipy* (1.3.1) for statistical analysis, and *matplotlib* (3.2.1) and *seaborn* (0.9.0) for visualizations.

Aggregated data underlying our main findings on threat perceptions of COVID-19 and influenza, confidence in organisations, and adoption rates of behaviours (broken down by country, sex, and age) are reported as CSV files in Additional file 3.

## Results

### Sample characteristics

Table 1 reports the participation rates and the characteristics of the sample per country, based on the unweighted sample. A total of 71,612 respondents completed the questionnaire in Belgium ($N=6253$), France ($N=6691$), Germany ($N=12\text{,}442$), Italy ($N=9741$), the Netherlands ($N=5292$), Spain ($N=7491$), the United Kingdom ($N=8753$), and the United States ($N=14\text{,}949$). Participation by week was high in all countries, ranging from a median number of 1114 respondents per week in the United Kingdom to 3058 in Germany. The sex ratio is somewhat skewed towards women compared to the overall population, ranging from 62% women in Germany to 70% women in France. Furthermore, older adults tend to be over-represented, with a median age ranging from 39 years (IQR 27-56) in Italy to 56 years (IQR 41-65) in the United Kingdom. As for educational attainment, there is some variation across countries. Most respondents attained university-level education in Belgium (47%), France (71%), Spain (60%), the United Kingdom (46%), and the United States (60%), whereas in Germany (61%), Italy (49%), and the Netherlands (58%) most respondents attained secondary-level education. See Supplementary Sect. 3, Additional file 1, for details on the sample after applying post-stratification weights.

**Table 1:** Characteristics of respondents who completed the COVID-19 Health Behavior Survey during the period March 13–April 19, 2020 in Belgium (BE), France (FR), Germany (DE), Italy (IT), Netherlands (NL), Spain (ES), United Kingdom (UK), and United States (US). Unweighted sample

	BE	FR	DE	IT	NL	ES	UK	US
No. participants	6253	6691	12,442	9741	5292	7491	8753	14,949
Participants per week								
Week 11 (March 9-15)	-	-	-	2016	-	-	1188	1583
				(21%)			(14%)	(11%)
Week 12 (March 16-22)	-	-	998	1937	-	-	800	2120
			(8%)	(20%)			(9%)	(14%)
Week 13 (March 23-29)	-	1374	1590	1849	-	1004	3148	3247
		(21%)	(13%)	(19%)		(13%)	(36%)	(22%)
Week 14 (March 30-April 5)	807	2356	3417	1771	1790	2458	1772	2873
	(13%)	(35%)	(27%)	(18%)	(34%)	(33%)	(20%)	(19%)
Week 15 (April 6-12)	3103	1605	3379	1085	1743	2404	1040	3088
	(50%)	(24%)	(27%)	(11%)	(33%)	(32%)	(12%)	(21%)
Week 16 (April 13-19)	2343	1356	3058	1083	1759	1625	805	2038
	(37%)	(20%)	(25%)	(11%)	(33%)	(22%)	(9%)	(14%)
Sex								
Female	4203	4712	7731	6337	3511	5128	5706	9833
	(67%)	(70%)	(62%)	(65%)	(66%)	(68%)	(65%)	(66%)
Male	2050	1979	4711	3404	1781	2363	3047	5116
	(33%)	(30%)	(38%)	(35%)	(34%)	(32%)	(35%)	(34%)
Age group								
18-24	1016	1203	2577	2001	705	561	686	1598
	(16%)	(18%)	(21%)	(21%)	(13%)	(7%)	(8%)	(11%)
25-44	1868	2086	4786	3949	1264	2743	2131	4051
	(30%)	(31%)	(38%)	(41%)	(24%)	(37%)	(24%)	(27%)
45-64	2174	2217	3391	2633	2004	3078	3626	5120
	(35%)	(33%)	(27%)	(27%)	(38%)	(41%)	(41%)	(34%)
65+	1195	1185	1688	1158	1319	1109	2310	4180
	(19%)	(18%)	(14%)	(12%)	(25%)	(15%)	(26%)	(28%)
Education								
Primary school or lower	298	127	189	354	215	296	97	22
	(5%)	(2%)	(2%)	(4%)	(4%)	(4%)	(1%)	(0%)
Secondary school	2717	1353	7635	4819	3079	1889	3254	5668
	(43%)	(20%)	(61%)	(49%)	(58%)	(25%)	(37%)	(38%)
University level	2919	4777	4036	4122	940	4465	4061	8989
	(47%)	(71%)	(32%)	(42%)	(18%)	(60%)	(46%)	(60%)
Other	319	434	582	446	1058	841	1341	270
	(5%)	(6%)	(5%)	(5%)	(20%)	(11%)	(15%)	(2%)

### Threat perceptions of COVID-19

**Figure 1:**
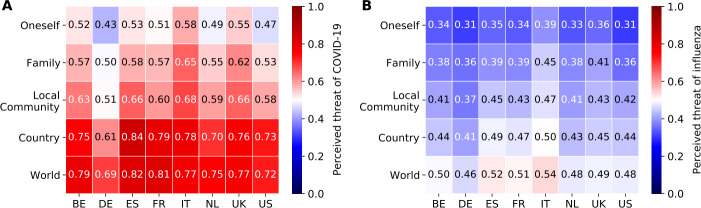
Comparison between the perceived threat posed by COVID-19 and influenza. Perceived threat posed by COVID-19 (**A**), and influenza (**B**) to oneself, the family, the local community, the country, and the world. The x-axis reports countries, namely Belgium (BE), France (FR), Germany (DE), Italy (IT), the Netherlands (NL), Spain (ES), the United Kingdom (UK), and the United States (US)

Figure 1A shows the threat that respondents perceived COVID-19 to pose to various levels of society. Overall, the threat perception is highest in Italy with a mean value of 0.69, followed by Spain with 0.68, the United Kingdom with 0.67, France with 0.66, Belgium with 0.65, the Netherlands with 0.62, the United States with 0.61, and lastly Germany with 0.55. To understand the meaning of these values, we compare them to the perceived threat posed by influenza (Fig. 1B). The heatmaps show that the perceived threat of COVID-19 is significantly higher than the perceived threat of the flu. In particular, the threat to oneself is on average 49% higher, the threat to the family is 46% higher, the threat to the local community is 45% higher, the threat to the country is 64% higher, and the threat to the world is 54% higher. More details about the perceived threat posed by influenza can be found in Supplementary Sect. 5, Additional file 1.

**Figure 2:**
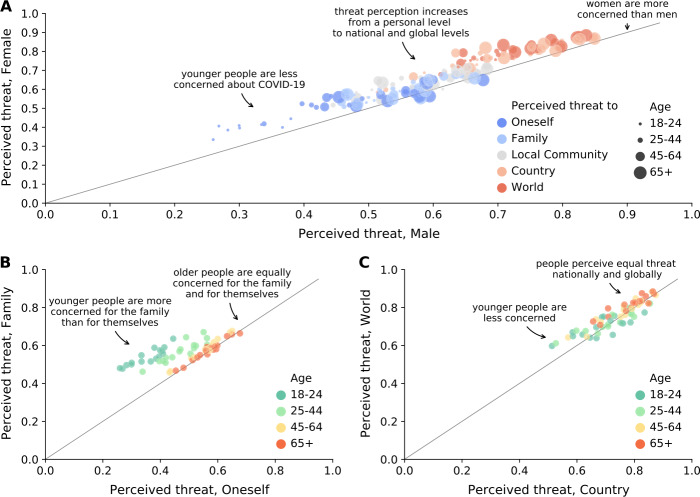
Threat perception of COVID-19. Relationship between the threat perceived by female and male respondents, where colours indicate the different levels of society and sizes indicate the age of respondents (**A**). Relationship between the threat perceived to oneself and to the family (**B**) and to the country and to the world (**C**) by age (indicated by the colour code)

Figure 2A shows the relationship between the threat perceived by male and female respondents for all levels of society and age groups. Here we observe several patterns. First, the perceived threat is significantly higher among women than among men, except for the threat to oneself and to the family among people aged 65 and over. Second, the perception of threat increases considerably from a personal level (threat to oneself and the family) to a national and global level (threat to the country and the world). Considering specifically the perceived threat to oneself and to the world, the latter is on average 51% greater. Third, younger people perceive lower threat compared to older people, except for the threat to their family. The latter finding is further supported by Figs. 2B and C, which show the relationship between the perceived threat to oneself and the family (Fig. 2B), and between the perceived threat to the country and the world (Fig. 2C), contingent on age. The youngest age group (i.e. 18-24) perceives a moderately low threat to themselves with a median value of 0.35 (IQR 0.34-0.38), but significantly higher to their family with a median value of 0.53 (IQR 0.52-0.55). By contrast, the oldest age group (i.e. 65+) perceives a moderately high threat both to themselves and to their family with a median value of 0.57 (IQR 0.53-0.60). On the other hand, the threat posed by COVID-19 at the national and global level is perceived similarly across all age group, while older individuals (45-64 and 65+) generally perceiving higher threat. See Supplementary Figure S9, Additional file 1, for a breakdown of threat perceptions by country, age, and sex.

**Figure 3:**
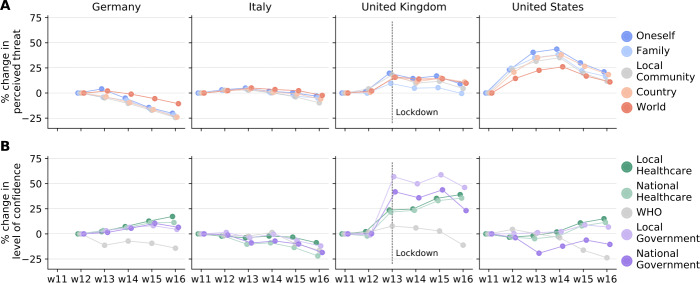
Temporal trend of COVID-19 threat perceptions and confidence in organisations. Weekly percent change in the perceived threat by COVID-19 (**A**) and in the level of confidence in organisations (**B**) in Germany, Italy, the United Kingdom, and the United States. The percent change is calculated by considering the initial value in the first week of data collection

Figure 3A shows the weekly percent change of threat perception compared to the initial value in Germany, Italy, the United Kingdom, and the United States. While in Germany the threat perception slowly decreased over time, in the United Kingdom and in the United States it drastically increased before decreasing to values closer to the ones of week 11. On the other hand, in Italy the threat posed by COVID-19 was rather constant over time.

### Confidence in organisations

**Figure 4:**
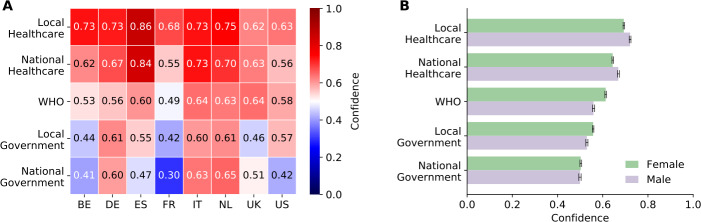
Confidence in organisations to deal with the COVID-19 pandemic. (**A**) Level of confidence in organisations, i.e. the local and national healthcare system, the World Health Organization (WHO), and the local and national government. The x-axis reports countries, namely Belgium (BE), France (FR), Germany (DE), Italy (IT), the Netherlands (NL), Spain (ES), the United Kingdom (UK), and the United States (US). Heatmap shows median values. (**B**) Level of confidence in organisations broken down by sex. Bar plot shows mean values and bootstrapped 95%CI as errors

Figure 4A shows the confidence that respondents have in the preparedness of various organisations to effectively deal with COVID-19. The confidence in the healthcare system is lowest in the United Kindgom and in the United States, and highest in Spain. In particular, people’s confidence in the national healthcare system tends to be lower than their confidence in the local healthcare system, except in Italy and in the United Kingdom (ranging from 2% lower in Spain to 19% lower in France). At the same time, the confidence in the government is lowest in France, while highest in Germany and the Netherlands. In particular, people report greater confidence in the national government in Italy, the Netherlands, and the United Kingdom (respectively 6%, 7%, and 13% higher than the confidence in the local government). In the remaining countries, people trust the national government less, ranging from 3% less in Germany to 28% less in France.

As Fig. 4B illustrates, men tend to be more confident in the local and national healthcare system, whereas women tend to be more confident in the WHO and the local government. No significant variation is observed in the confidence in the national government, although there is a substantial difference in the United States, where men have greater confidence in the national government than women. See Supplementary Figure S10, Additional file 1, for a breakdown of confidence ratings by country, age group, and sex.

Figure 3B shows the weekly percent change in confidence compared to the initial value in the first week of data collection in Germany, Italy, the United Kingdom, and the United States. Across countries, people lose trust in the WHO over time. In Germany, the confidence in both the healthcare system and the government shows a positive trend. Similarly, in the United Kingdom confidence drastically increased after the government decision of locking down the country. In Italy, people’s confidence slowly decreased, and it was approximately 16% lower in week 16 compared to week 11. In the United States, by contrast, the temporal pattern is more variable. The confidence in the healthcare system and the local government increased from week 15, after remaining constant for nearly a month, whereas people’s confidence in the national government decreased and remained below the initial value.

### Preventive behaviours

**Figure 5:**
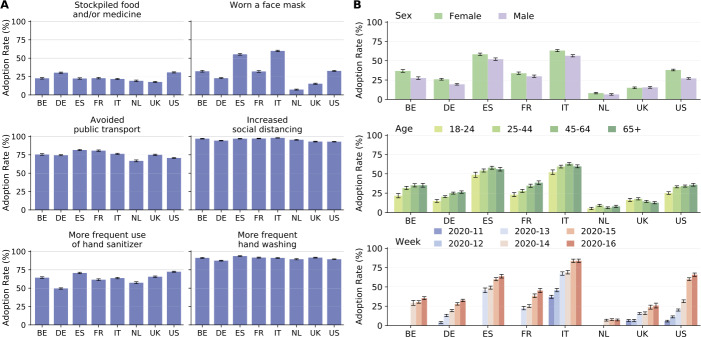
Adoption of preventive behaviours. (**A**) Adoption rate of behaviours by country defined as the weighted proportion of individuals who adopted a specific behaviour. (**B**) Adoption rate of wearing a face mask, by sex (top), age (center), and calendar week (bottom). Bar charts show mean values as bars and 95%CI as errors

Figure 5A shows the adoption rate of behaviours by country during the period March 13–April 19, 2020. The least frequent behaviour is the stockpiling of food and/or medicine, ranging from about 18% in the United Kingdom to about 31% in the United States. Wearing a face mask ranges from about 7% in the Netherlands to about 60% in Italy. As for hand hygiene, the adoption of more frequent use of hand sanitizer ranges from about 50% in Germany to about 72% in the United States, whereas the adoption of more frequent hand washing ranges from about 87% in Germany to about 94% in Spain. The most frequently reported behaviours are, respectively, a reduced use of transportation, ranging from about 67% in the Netherlands to about 82% in Spain, and increased social distancing, ranging from about 93% in the United States to about 98% in Italy.

Figure 5B shows the adoption rate of wearing a protective face mask by sex, age, and calendar week. Apart from the Netherlands and the United Kingdom, women and people aged 45 years and over show the highest adoption rates of face masks. Moreover, the use of a face mask substantially increased over time, except in Belgium and in the Netherlands. Note that in the observation period protective face masks were not yet mandatory, but still rapidly adopted in the population.

Supplementary Figure S11, Additional file 1, shows the adoption of the remaining behaviours by age, sex, and calendar week. On average, women tend to adopt more protective behaviours than men. Social distancing has increased sharply in the United Kingdom and in the United States, whereas it has decreased in Germany, reflecting different stages of the epidemic and different policies.

## Discussion

Understanding how different demographic groups perceive the risk of COVID-19, and consequently adopt protective behaviours, is key to increase situational awareness and inform policy makers in designing optimal intervention strategies. Here, we have presented insights from survey data collected through a cross-national online survey, the COVID-19 Health Behavior Survey (CHBS), during the period March 13–April 19, 2020. To the best of our knowledge, our study provides the most comprehensive and rigorous cross-national and comparative data during the peak months of the first wave of the COVID-19 pandemic in Europe and the United States. In this section, we summarize our findings and provide our interpretation in light of the current evidence on the pandemic.

First, we found substantial spatio-temporal heterogeneity in behaviours and attitudes between countries that were in different stages of the pandemic and with different control strategies in place. In Europe, Italy was the first country most affected by COVID-19 and the first to order a nationwide lockdown on March 11, 2020. This may explain the high threat perception of COVID-19, and, together with the high confidence in healthcare systems and governments, the willingness to adhere to the recommended measures. Similar lockdowns were then implemented in Spain (March 14), France (March 17), Belgium (March 18), Germany (March 22), the Netherlands (March 24), and the United Kingdom (March 24), while statewide restrictive measures were implemented in the United States, starting in California on March 19, 2020. In the United Kingdom and the United States (as a whole) we observe substantial temporal variation before and after government decisions, with the perceived threat increasing over time, along with a rapid adoption of social distancing measures and reduced mobility. By contrast, a somewhat less restrictive lockdown was implemented in Germany, allowing outdoor activities for families and people living in the same household. Nonetheless, the adoption of social distancing measures was high from the very beginning of our observation period, as well as the confidence in the healthcare system and government, which has further increased over time. At the same time the perceived threat of COVID-19 has decreased. Indeed, of the European countries considered in this study, Germany was ranked third in terms of infections (about 140,000), but only sixth in terms of deaths (about 4000) as of April 19, 2020, which may explain the lower risk perceived by the population [19]. Furthermore, we captured a crucial behavioural change in the population in the use of protective face masks, which grew rapidly at a time when they were not mandatory yet. This is an important finding, especially in contemporary Western societies where, in the early phase of the pandemic, wearing face protective masks amongst the general public could be interpreted as a case of bottom-up behavioural change.

Second, we observe a clear pattern in threat perceptions of COVID-19 sharply increasing from moderate threat at the personal level (oneself and the family) to high threat at the national and global levels. Perceptions of personal threat may be an indicator of adopting protective behaviours. However, although personal threat perceptions were comparatively low among our respondents, we found significant increase in hand hygiene. This renders it uncertain as to what extent behaviour can be straightforwardly linked to perceptions of personal threat. Furthermore, we found that threat perceptions of COVID-19 were significantly higher than threat perceptions of influenza. Yet, this is not surprising given the novelty and uncertainty surrounding COVID-19.

Third, we found age-specific differences, with older people perceiving higher threat to themselves. On the other hand, all respondents were strongly concerned about their family members regardless of their own age and the perceived threat to themselves. This is in line with the evidence that older adults are at highest risk of severe complications following infection from COVID-19 [20].

Fourth, we also found gender-specific patterns, with women reporting higher threat perceptions of COVID-19, lower confidence in the healthcare system, and higher adoption of protective behaviours than men. This finding is in line with a recent study that showed that women are more likely to perceive the pandemic as a very serious health problem and to agree and comply with restraining measures [21]. Since the case fatality rate for COVID-19 is substantially higher for men [22], this evidence is relevant for policy makers in designing communication campaigns on COVID-19, which may need to be gender based in order to tackle this difference in attitudes and behaviours.

In this study, we used targeted Facebook advertisements for participant recruitment. Previous studies have highlighted the benefits of using Facebook for online surveys in demographic and health research [14, 23, 24]. Facebook is currently the largest social media platform, with 2.45 billion monthly active users worldwide as of September 2019 and high penetration rates, ranging from 56% in Germany to 92% in Denmark and about 69% in the United States [25–27]. However, compared to previous studies, our survey stands out because of its timeliness, cross-national and comparative nature, and population coverage in an exceptional situation like a pandemic [15].

These advantages notwithstanding, our approach also has some limitations. First, online surveys potentially suffer from bias due to self-selection and non-representativeness of the sample. In the case of Facebook, there is increasing evidence that samples obtained from this social media network are not significantly different in central demographic and psychometric characteristics from samples obtained from more traditional recruitment and sampling techniques [9]. Furthermore, by applying post-stratification weighting we can correct for non-representativeness at least in central observable characteristics. Ideally, we would apply this approach on a weekly basis to warrant complete comparability of observations over time, but data scarcity issues complicate this approach. However, we do not expect this to strongly affect our results. Similarly, self-selection of online survey respondents as well as under-representation of minority groups represent potential issues which cannot be corrected by our post-stratification approach. The limited language availability in both the ads and surveys in our study, for example, may have triggered under-representation of specific groups. However, the number of respondents who completed the questionnaire in a language other than the one in which they landed at the survey page is very low (less than ten respondents per country), whereas the sample of foreign born in our dataset is substantial, ranging from 6% in the United States to 22% in Spain, thus making our sample diverse enough not to strongly affect our results. More methodological work to assess biases is beyond the scope of this article, but we consider it an important and promising line of research to further advance the field and to guarantee appropriate coverage of under-represented groups.

Second, given the cross-sectional nature of the study and the lack of a baseline for pre-pandemic behaviours, our survey data enable us to assess changes in the population samples over time, and capture behavioural changes at the individual level only to a limited extent. In the near future, we will carry out a follow-up survey among those respondents who agreed to provide their email address and to be contacted again for similar surveys. This panel perspective will offer a unique possibility to study the long-term impact of the COVID-19 pandemic on the population in a cross-national perspective.

## Conclusions

Our work reduces the gap in human behavioural data at a time when the need for timely and key data is key to informing interventions. Our findings are relevant for decision makers in designing appropriate public health campaigns, and for researchers in modelling more realistic epidemic approaches for scenario analysis, accounting for accurate data on human behaviours. Our work also illustrates how social media networks, like Facebook, together with appropriate survey designs and statistical methods, offer an innovative and powerful tool for rapid and continuous data collection to monitor trends in behaviours relevant for mitigation strategies of COVID-19.

## Supplementary Information

Below are the links to the electronic supplementary material.

Supplementary materials, including separate sections on Facebook advertising campaigns, response rates, post-stratification approach, respondent selection, and threat perception of influenza. (PDF 3.2 MB)

The English version of the questionnaire used in the study. (PDF 411 kB)

Aggregated data of the variables presented in this study, i.e. threat perceptions of COVID-19, threat perceptions of influenza, confidence in organisations, and adoption rates of behaviours. (ZIP 24 kB)

## Data Availability

The datasets collected and analysed in this study are not publicly available due to data protection policy. The aggregated data underlying the main findings in this study are reported in the Supplementary Information.
