# Supplementary material for: Behaviours and attitudes in response to the COVID-19 pandemic: insights from a cross-national Facebook survey
Source: EPJ Data Sci. 2021 Apr 14;10(1):17. doi: 10.1140/epjds/s13688-021-00270-1 (PMC8050509; doi:10.1140/epjds/s13688-021-00270-1)
Supplement: Supplementary file 1 — Supplementary materials, including separate sections on Facebook advertising campaigns, response rates, post-stratification approach, respondent selection, and threat perception of influenza. (PDF 3.2 MB) [file 13688_2021_270_MOESM1_ESM.pdf]

Supplementary Material

Behaviours and attitudes in response to the COVID-19 pandemic: Insights from a cross-national Facebook survey

Daniela Perrotta[1], André Grow[1], Francesco Rampazzo[2], Jorge Cimentada[1], Emanuele Del Fava[1], Sofia Gil-Clavel[1], and Emilio Zagheni[1]

[1]Max Planck Institute for Demographic Research, Germany
[2]Saïd Business School, Leverhulme Centre for Demographic Science, and Nuffield College, University of Oxford, Oxford, United Kingdom

1 Facebook advertising campaigns

Facebook advertising campaigns consist of three layers: (i) the campaign level, at which the goals of the campaign are specified (generating impression, generating clicks, etc.); (ii) the ad set level, at which several target groups can be specified (one target group per ad set); (iii) the ad level, at which the advertising text and the images that are to be used can be specified. As indicated in the main part of the paper, we created one advertising campaign for each country, and each campaign was stratified by sex, age, and region, so that there was one ad set per stratum, each with six advertisements that differed only in the picture that was used. Figure S1 illustrates this for the United States, and Table S4 shows the mapping of the detailed regions of residence onto the macro-regions that we used in the campaigns.

We opted for this approach because the FAM uses algorithms that optimize the return for advertisers and this may lead to biased samples. For example, if the goal of a campaign is to generate ad clicks, the FAM will over time increasingly show a given ad to user groups who are most likely to click on it[1]. This can lead to a biased sample, if members of certain demographic groups are more likely to click on the ad than members of other groups. To avoid this problem, we followed the recommendations of Pötzschke and Braun[1] and created one ad set for each stratum of the population, thereby ensuring a more balanced delivery of the ads. The selected campaign goal was generating clicks, and we used six different ad images, to ensure that our ads appeal to a wide audience.

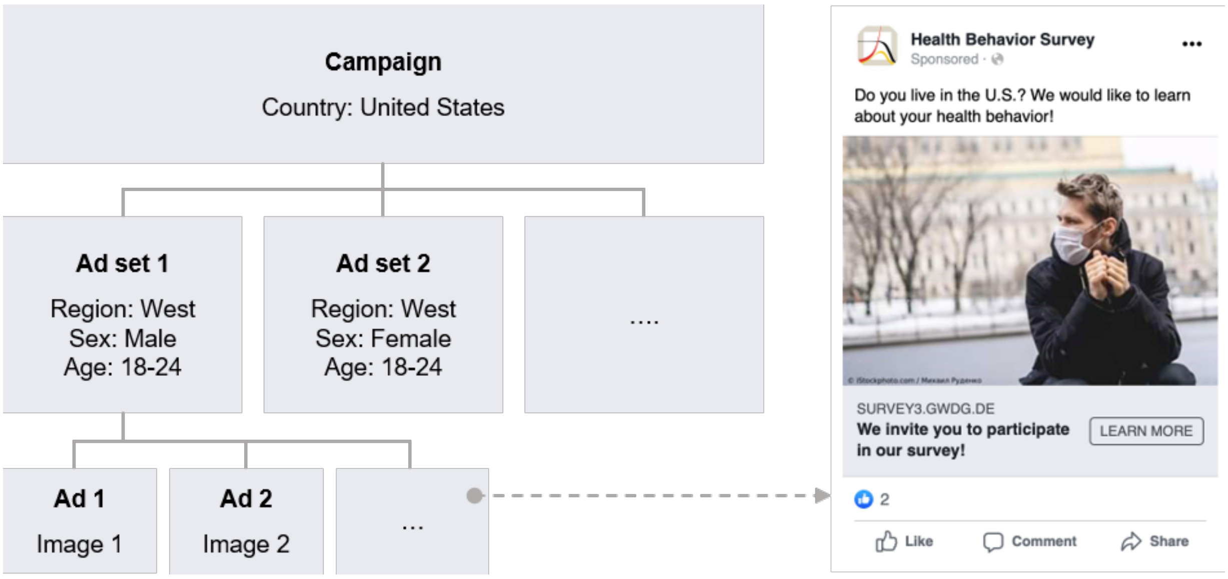

Fig. S1. Design of Facebook advertising campaigns. Illustration of the structure of the advertising campaign used in the United States (left). Adapted from Figure 2 in Pötzschke and Braun[1]. Example of an advertisement shown to Facebook users in the United States (right).

2 Response rates

In the context of online surveys, it is often difficult to calculate traditional performance measures, such as the response rate, because the number of members of the target population who have actually seen an invitation to participate in the survey cannot be determined[2]. Our use of the FAM enables us to address this issue. The FAM provides estimates of how many users have seen an advertisement at least once, and how many of them have clicked on the advertisement

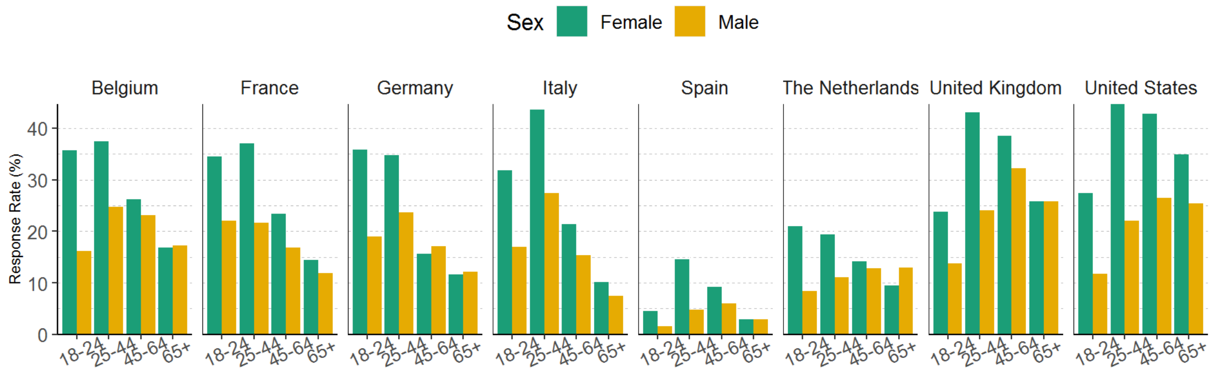

Fig. S2. Facebook response rate. Response rate by age and sex per each country in the study, in the period between March 13, 2020 and April 19, 2020.

at least once. In combination with information about the number of completed questionnaires, this information makes it possible to calculate approximate participation rates. Specifically, we calculated the response rate as the ratio of the number of completed questionnaires (considering only participants who were directly targeted by one of our advertisement) and the number of unique Facebook users who clicked on the advertisement, according to Facebook.

Figure S2 shows the participation rates by country, sex, and age group. The overall response rate in the period March 13 to April 19, 2020 was approximately 25% in Belgium, 22% in France, 21% in Germany, 21% in Italy, 6% in Spain, 14% in the Netherlands, 6% in Spain, 31% in the United Kingdom, and 31% in the United States. Female users were generally more likely to complete the questionnaire compared to male users. Across countries, the participation rate varies across sex and age groups; for example, in Italy it ranged from about 8% (Italy, men age 65+) to about 43% (Italy, women age 25-44). The only exception from this is Spain, where participation rates were considerably lower, ranging from about 2% (men age 18-24) to about 15% (women age 25-44).

3 Post-stratification

In this study, we use a post-stratification weighting approach to correct for potential issues with non-representativeness in the sample. This is a standard procedure in survey research, in which appropriate weights are computed based on population information from representative data sources (e.g. census data). Here, we use population data from Eurostat (2019)[3] and the US census (2018)[4].

Table S1 shows an example of the post-stratification approach for the strata in the macro-region 'Central' in Italy (see Table S4 for the corresponding micro-regions), presenting both the Facebook survey counts and proportions and the true population counts and proportions obtained from Eurostat. The columns 'Region', 'Sex' and 'Age' together define the eight selected strata. The columns Facebook $\hat{N}_i$ and Facebook $\hat{p}_i$ respectively show the numbers of respondents belonging to each stratum and their proportions relative to all strata (note that these proportions do not sum up to 1 because they are relative to all strata, including the ones not presented here). Conversely, the columns Eurostat N_i and Eurostat p_i show the number of people from the true population that belong to that stratum and their proportion relative to the complete population. The weights w_i are then defined as $w_i = p_i/\hat{p}_i$.

Since the official population counts from Eurostat (as well as the US census) are provided

Table S1. Example of post-stratification reweigh using the central region of Italy.

			Facebook		Eurostat		
Region	Sex	Age	$\hat{N}_i$	$\hat{p}_i$	N_i	p_i	w_i
Central	Female	18-24	199	0.027	522,730	0.010	0.363
Central	Female	25-44	384	0.053	1,453,810	0.028	0.523
Central	Female	45-64	265	0.037	1,876,591	0.036	0.979
Central	Female	65+	104	0.014	1,605,018	0.031	2.133
Central	Male	18-24	119	0.016	572,911	0.011	0.665
Central	Male	25-44	214	0.030	1,454,223	0.028	0.939
Central	Male	45-64	176	0.024	1,758,096	0.034	1.380
Central	Male	65+	78	0.011	1,221,951	0.023	2.165

in five-year age groups (e.g. 15-19 years, 20-24 years, ...), whereas we required respondents to be at least 18 years old, the true population counts for the 18-24 age group is not directly comparable between our survey counts and the Eurostat/US census population counts. To address this issue, we multiply the Eurostat/US census population counts for the age group 15-24 by 7/10, reflecting that we only have seven of the ten ages in that age group. As a consequence, there is more uncertainty for the estimates in that age group.

Figure S3 shows the unweighted and weighted Facebook estimates by sex, age groups, and education, in comparison with nationally representative surveys, respectively, the European Social Survey (ESS, 2018[5]) and the American Community Survey (ACS, 2018[6]). For illustration, we focus here on Italy; the same interpretation applies to the remaining countries. The top row of panels show how the unweighted and weighted estimates from our survey differ from the ESS estimates for Italy. For example, in the leftmost top panel (sex for Italy) the ESS proportions estimate that there are 51% CI [49%, 53%] female respondents and 49% CI [47%, 51%] male respondents in Italy. In our *unweighted* sample, there are 65% female respondents and 35% male respondents, a bias of around 14% points. However, our *weighted* sample corresponds very closely with the ESS estimates. Female respondents are now estimated to be 52% CI [51%, 53%] of the sample and male respondents 48% CI [47%, 49%] of the sample. The same trend can be seen for the age groups (center plot for Italy). For example, the unweighted percentage of the age group 18-24 in our sample is 20%, whereas the weighted ESS estimate is 10% CI [8%, 11%]. Nonetheless, after reweighing, the estimate of the 18-24 age group in our survey is now 11% CI [11%, 12%], very close to the benchmark of the ESS. The last example comes from the education variable, where the counts are more similar between the unweighted and weighted estimates. We might expect that the educational composition of our sample is more biased than its composition in terms of age and sex variables, given that we did not stratify our advertising campaigns based on this variable. However, the distribution of educational attainment in our sample resembles the distribution from the ESS, with the exception that our unweighted sample under-counts the proportion of primary and secondary educated respondents, and over-counts the respondents with graduate education. This bias differs across countries, with France showing the largest deviations and Germany the smallest deviations.

Taken together, our results suggest that when appropriately weighting the different strata according to reliable population counts, the initial bias in our sample can be adjusted, so that our data better reflect the general population. However, the adjustment is not perfect as can be seen from the distribution of the education variable and other variables, where our weighted estimate differs from the ESS estimates.

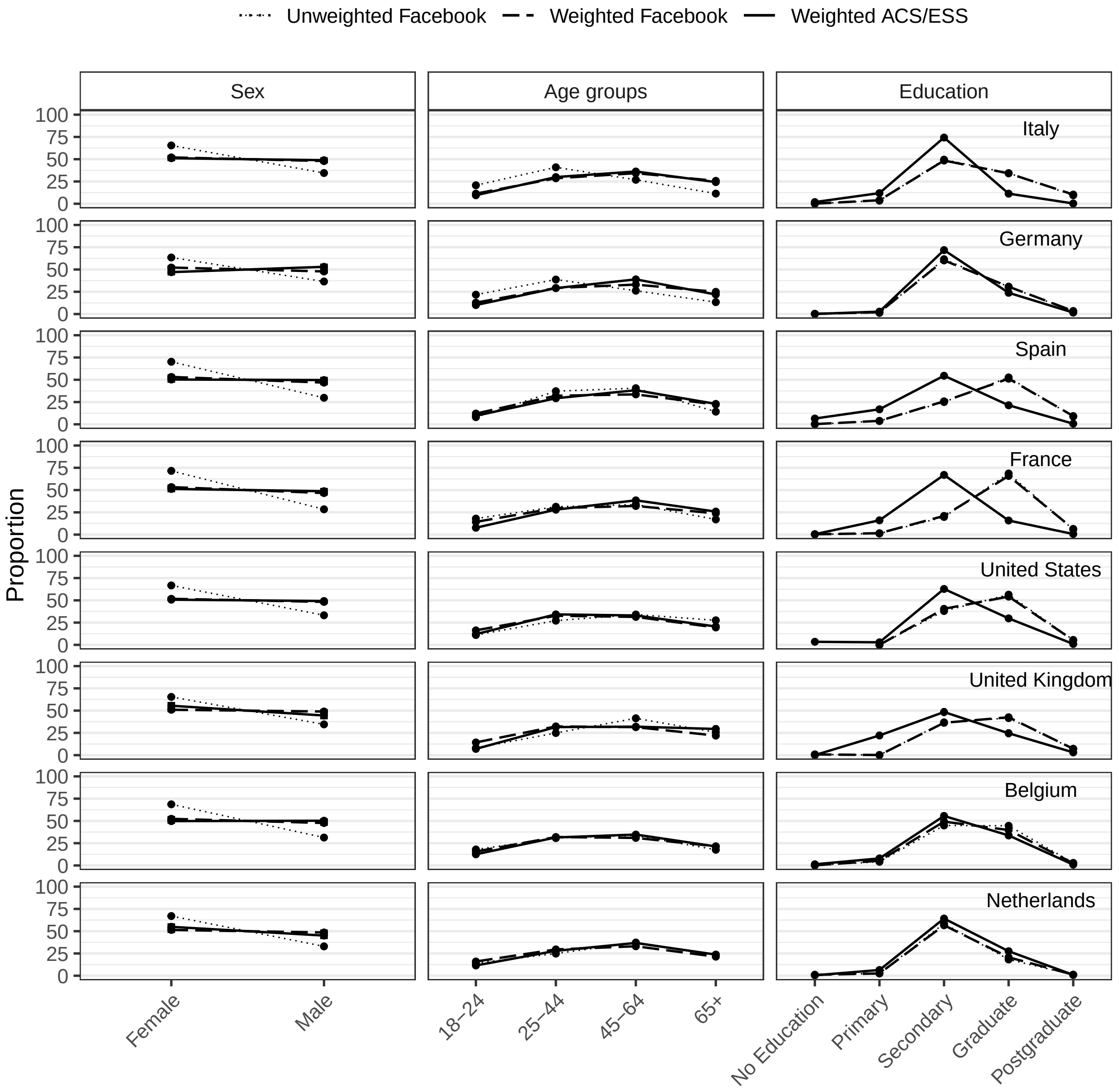

Fig. S3. Post-stratification weighting approach. Comparison of main demographic variables before and after post-stratification reweighing with estimates from the European Social Survey (ESS) and the American Community Survey (ACS) for Italy, Germany, Spain, France, United States, United Kingdom, Belgium, and Netherlands.

4 Respondent selection

As we describe in the main part of the paper, only respondents who indicated that they were at least somewhat aware of the coronavirus outbreak were asked questions related to it. Table S2 shows the number of respondents per answer category in the corresponding filter question by country. Those who chose "Nothing at all" or "Prefer not to answer" were excluded. Furthermore, in the analysis, we excluded respondents item-by-item. Table S3 shows the number of respondents that had be excluded for each of the questions that we considered by country.

Table S2. Level of participants' awareness of the coronavirus outbreak. Columns refer to countries, namely Belgium (BE), France (FR), Germany (DE), Italy (IT), the Netherlands (NL), Spain (ES), the United Kingdom (UK), and the United States (US). Unweighted sample.

Awareness	BE	FR	DE	IT	NL	ES	UK	US
A great deal	5,304 (84%)	6,394 (94%)	8,835 (70%)	8,544 (87%)	3,602 (67%)	5,644 (73%)	7,414 (84%)	13,295 (89%)
A fair amount	860 (14%)	252 (4%)	3,036 (24%)	1,175 (12%)	1,644 (31%)	1,895 (25%)	1,325 (15%)	1,617 (11%)
Not very much	131 (2%)	90 (1%)	627 (5%)	45 (0.5%)	94 (2%)	114 (1%)	51 (0.6%)	67 (0.4%)
Nothing at all	15 (0.2%)	21 (0.3%)	47 (0.4%)	9 (0.1%)	9 (0.2%)	10 (0.1%)	5 (0.1%)	9 (0.1%)
Prefer not to answer	26 (0.4%)	38 (0.6%)	57 (0.5%)	20 (0.2%)	14 (0.3%)	41 (0.5%)	15 (0.2%)	16 (0.1%)

Table S3. Participants who selected "Don't know" or "Prefer not to answer" in the threat perception of COVID-19 and levels of confidence. Columns refer to countries, namely Belgium (BE), France (FR), Germany (DE), Italy (IT), the Netherlands (NL), Spain (ES), the United Kingdom (UK), and the United States (US). Unweighted sample.

	BE	FR	DE	IT	NL	ES	UK	US
Perceived threat								
Oneself	122 (2%)	190 (3%)	135 (1%)	150 (2%)	133 (3%)	214 (3%)	163 (2%)	207 (1%)
Family	204 (3%)	235 (4%)	296 (2%)	165 (2%)	192 (4%)	243 (3%)	243 (3%)	344 (2%)
Local Community	177 (3%)	206 (3%)	212 (2%)	92 (1%)	126 (2%)	92 (1%)	140 (2%)	160 (1%)
Country	61 (1%)	81 (1%)	134 (1%)	63 (1%)	86 (2%)	54 (1%)	65 (1%)	110 (1%)
World	95 (2%)	109 (2%)	193 (2%)	101 (1%)	115 (2%)	85 (1%)	85 (1%)	192 (1%)
Level of confidence								
Local Health System	248 (4%)	303 (5%)	740 (6%)	172 (2%)	345 (7%)	110 (2%)	326 (4%)	440 (3%)
National Health System	221 (4%)	191 (3%)	522 (4%)	95 (1%)	230 (4%)	85 (1%)	136 (2%)	269 (2%)
WHO	310 (5%)	335 (5%)	840 (7%)	279 (3%)	399 (8%)	207 (3%)	383 (4%)	674 (5%)
Local Government	454 (7%)	606 (9%)	895 (7%)	194 (2%)	540 (10%)	215 (3%)	855 (10%)	338 (2%)
National Government	222 (4%)	152 (2%)	302 (2%)	163 (2%)	248 (5%)	160 (2%)	125 (1%)	231 (2%)

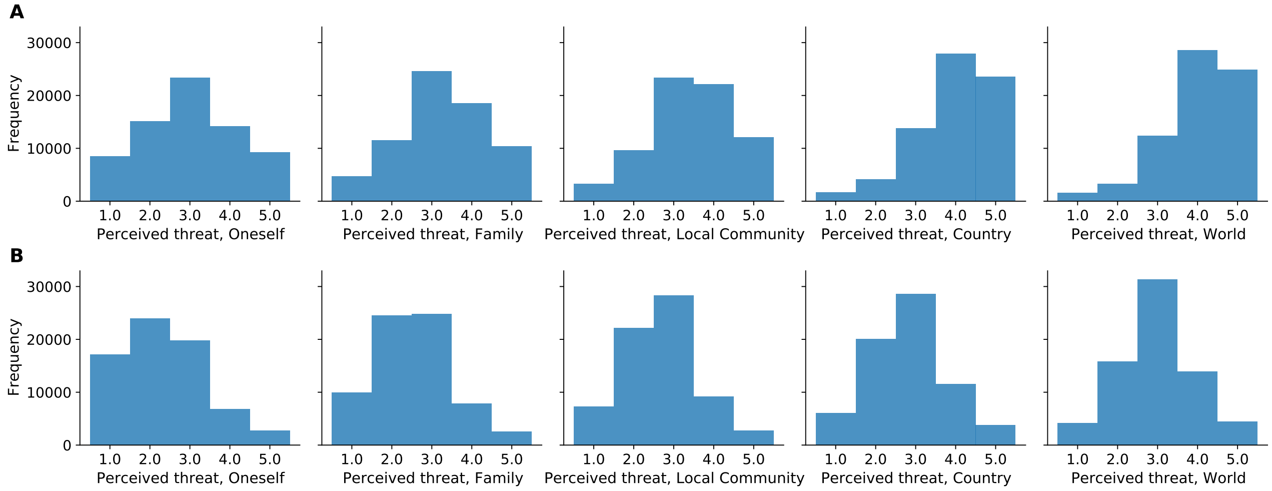

Fig. S4. Distributions of threat perceptions of COVID-19 and influenza. Perceived threat posed by COVID-19 (**A**) and influenza (**B**) to oneself, the family, the local community, the country, and the world. Responses reported on a 5-point Likert-type scale (1 = very low threat, 5 = very high threat). Weighted sample.

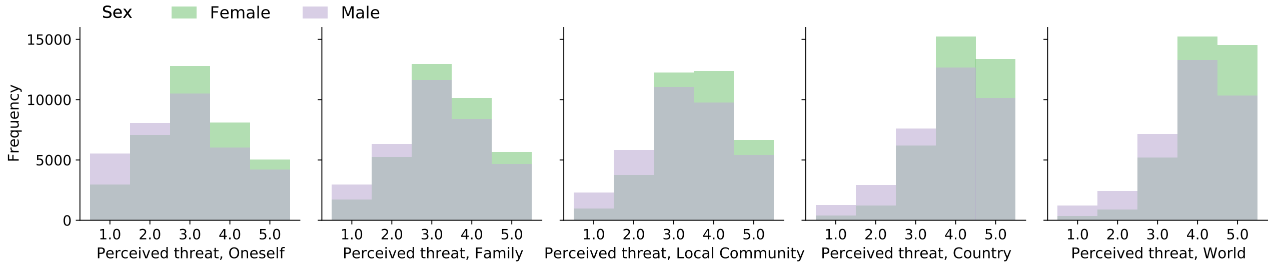

Fig. S5. Distribution of threat perception of COVID-19 by sex. Perceived threat posed by COVID-19 to oneself, the family, the local community, the country, and the world, broken down by sex. Responses reported on a 5-point Likert-type scale (1 = very low threat, 5 = very high threat). Weighted sample.

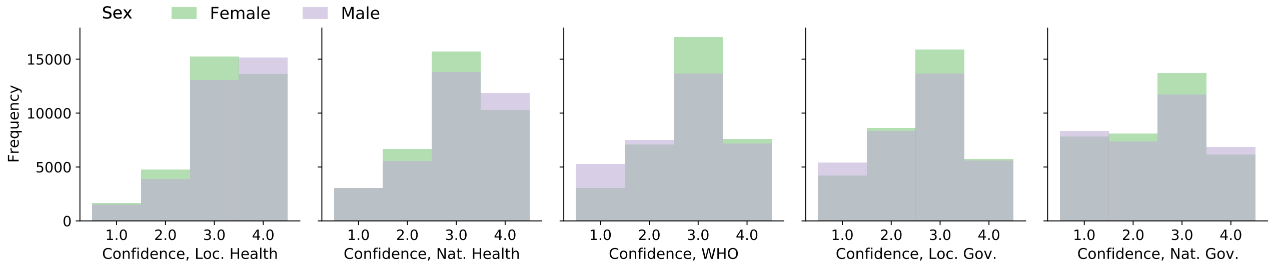

Fig. S6. Distribution of confidence in organisations by sex. Level of confidence in the local and national healthcare system, the World Health Organization (WHO), and the local and national government, broken down by sex. Responses reported on a 4-point Likert-type scale (1 = not confident at all, 4 = very confident). Weighted sample.

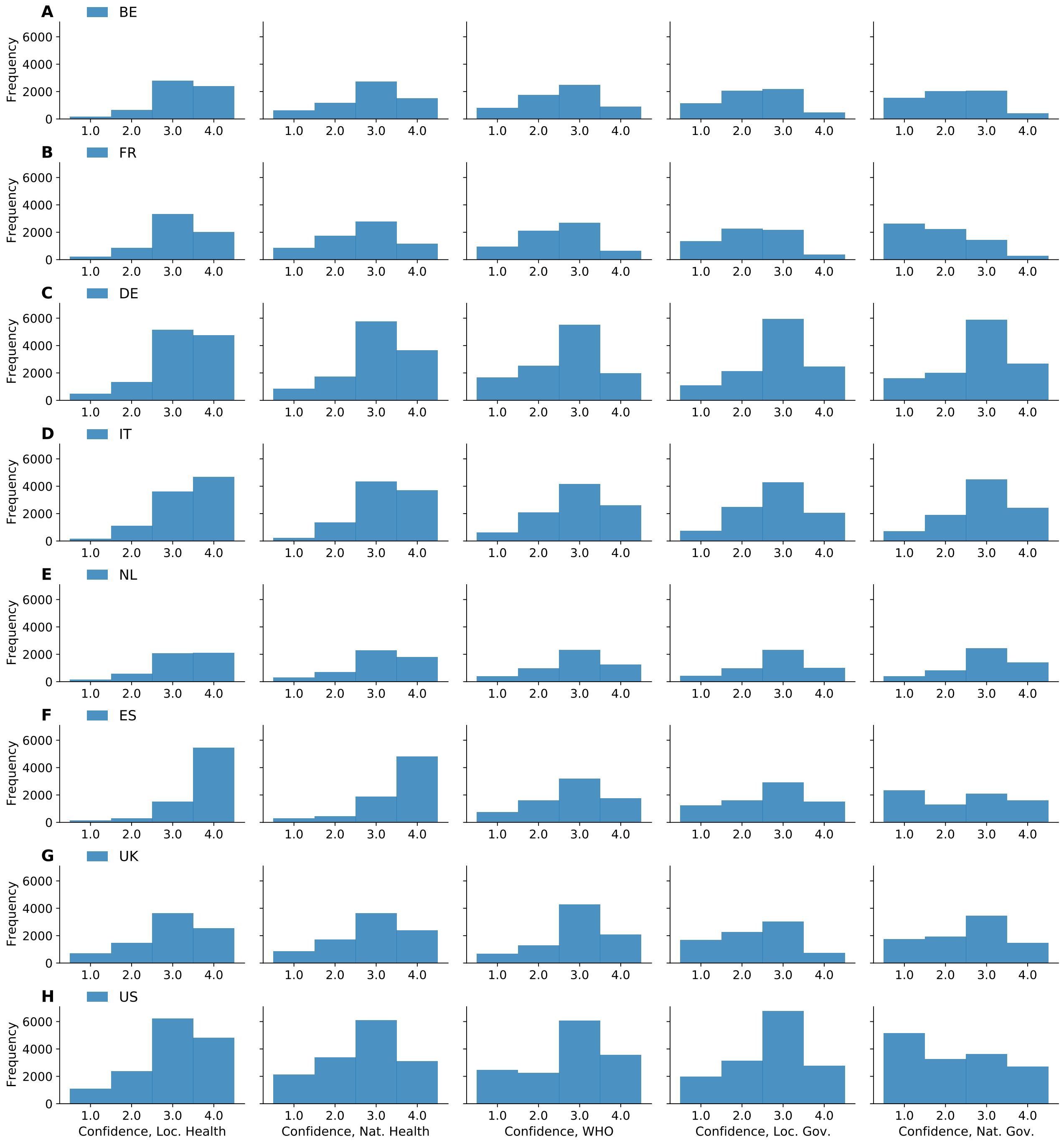

Fig. S7. Distribution of confidence in organisations by country. Level of confidence in the local and national healthcare system, the World Health Organization (WHO), and the local and national government, broken down by country, i.e. Belgium (**A**), France (**B**), Germany (**C**), Italy (**D**), the Netherlands (**E**), Spain (**F**), the United Kingdom (**G**), and the United States (**H**). Responses reported on a 4-point Likert-type scale (1 = not confident at all, 4 = very confident). Weighted sample.

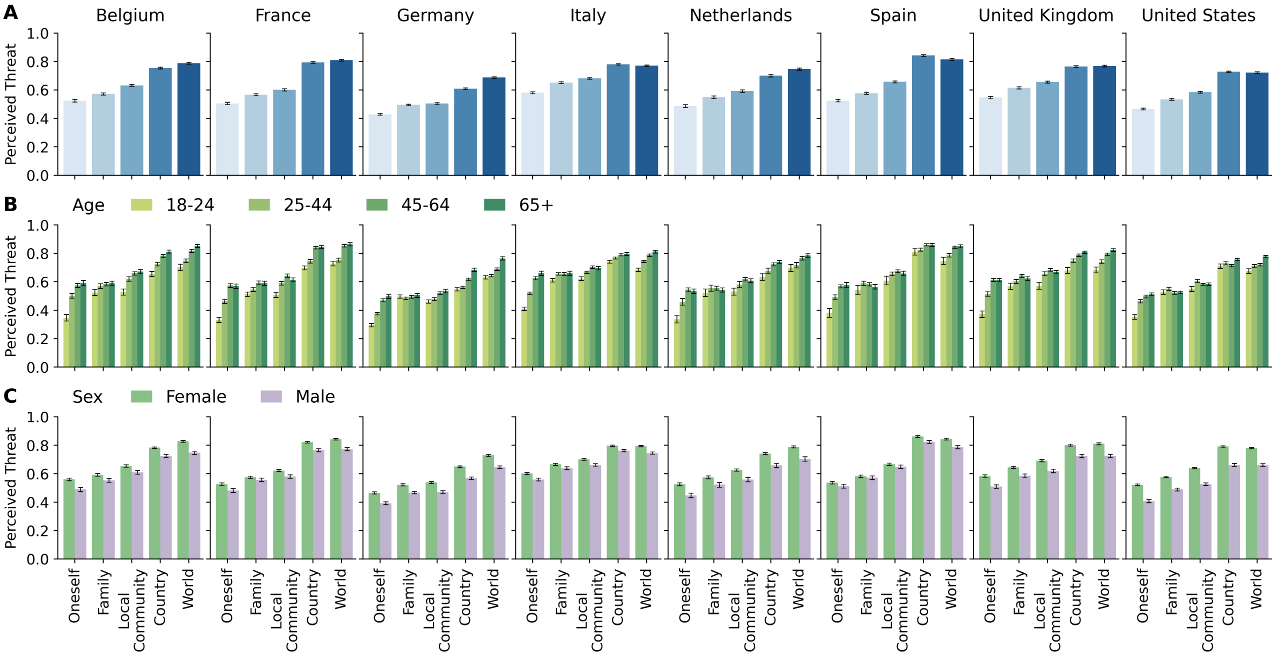

Fig. S8. Perception of threat posed by influenza. Perceived threat posed by influenza to oneself, the family, the local community, the country, and the world, broken down by country (**A**), age group (**B**), and sex (**C**). Bar charts show mean values as bars and bootstrapped 95%CI as errors. Weighted sample.

5 The threat perception of influenza

Figure S8 shows the threat that respondents perceived influenza to pose for different levels of society (i.e. to oneself, the family, the local community, the country, and the world), broken down by country (panel A), age group (panel B), and sex (panel C). Overall, threat perception of influenza is highest in Italy with a median value of 0.47, followed by Spain with 0.44, the United Kingdom with 0.43, France with 0.43, Belgium with 0.41, the Netherlands with 0.41, the United States with 0.40, and lastly Germany with 0.38.

As for the threat perception of COVDI-19, the threat perception of influenza increases sharply from the personal sphere (oneself and the family) to more distal contexts, i.e. the local community, the country, and, ultimately, the world. Considering specifically the perceived threat to oneself and to the world, the latter is on average 47% greater, whereas this difference ranges from 37% in Italy to 56% in the United States.

Moreover, the perceived threat posed by influenza is both age- and sex-specific. As shown in Figure S8B, the perceived threat sharply increases with age (all $p < 0.004$, except the threat to the family [$p = 0.1$]). In particular, the perceived threat to oneself is consistently lower for the younger age group (i.e. 18-24) with a mean value of 0.19 (IQR 0.18-0.21), and higher for the elderly, i.e. 65+, with a mean value of 0.42 (IQR 0.41-0.44). On the other hand, when it comes to the family, younger people perceive a higher threat, with a mean value of 0.32 (IQR 0.30-0.33), while it remains unvaried for the elderly. Furthermore, as Figure S8C shows, the perceived threat is significantly higher among female respondents than among male respondents (all $p < 0.001$).

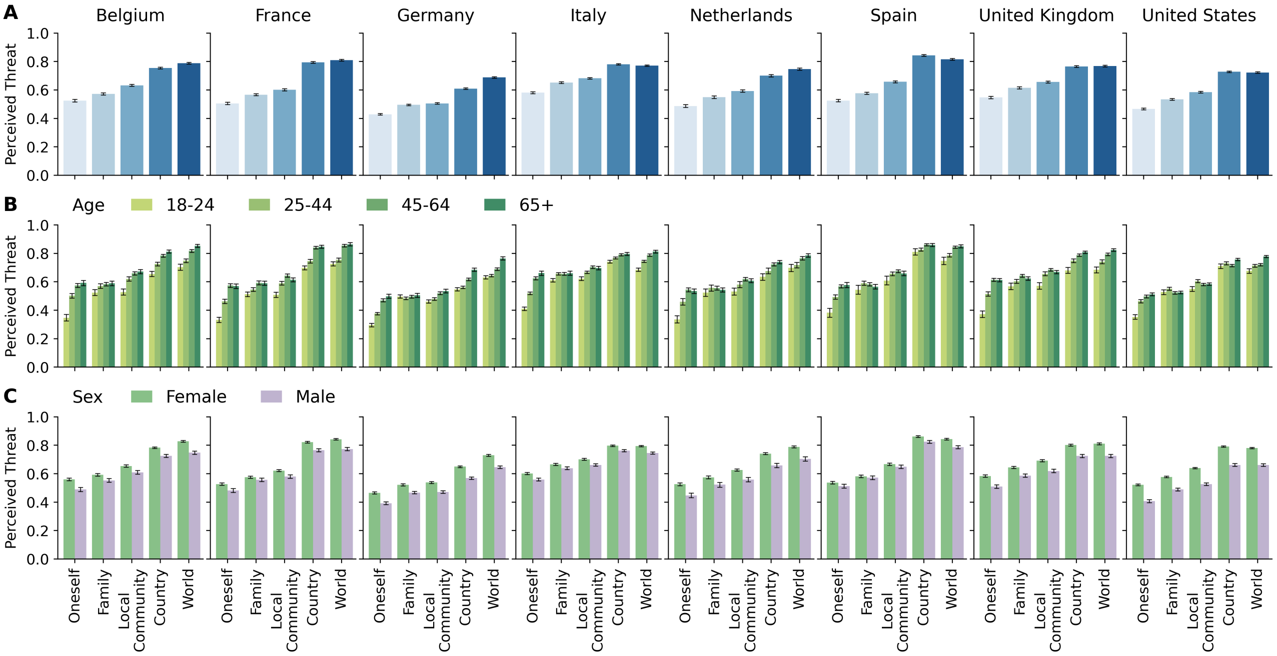

Fig. S9. Perception of threat posed by COVID-19. Perceived threat posed by COVID-19 to oneself, the family, the local community, the country, and the world, broken down by country (**A**), age group (**B**), and sex (**C**). Bar charts show mean values as bars and bootstrapped 95%CI as errors. Weighted sample.

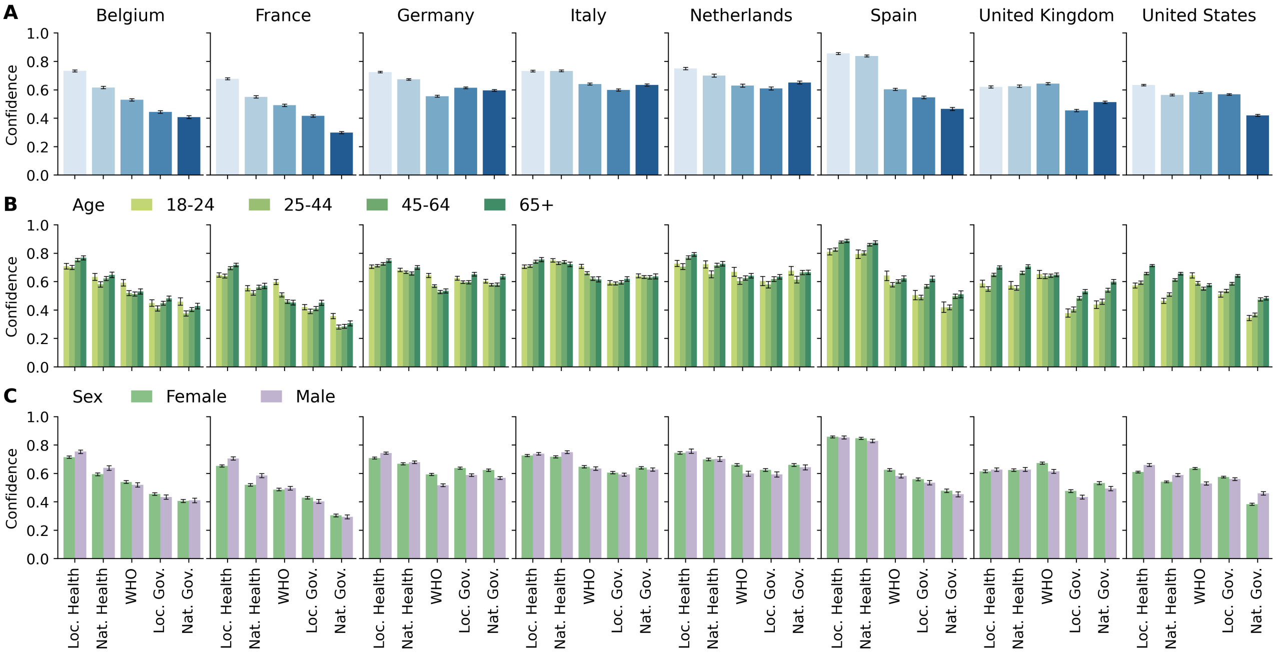

Fig. S10. Confidence in organisations to deal with the COVID-19 pandemic. Level of confidence in the local and national healthcare system, the World Health Organization (WHO), and the local and national government, broken down by country (**A**), age group (**B**), and sex (**C**). Bar charts show mean values as bars and bootstrapped 95%CI as errors. Weighted sample.

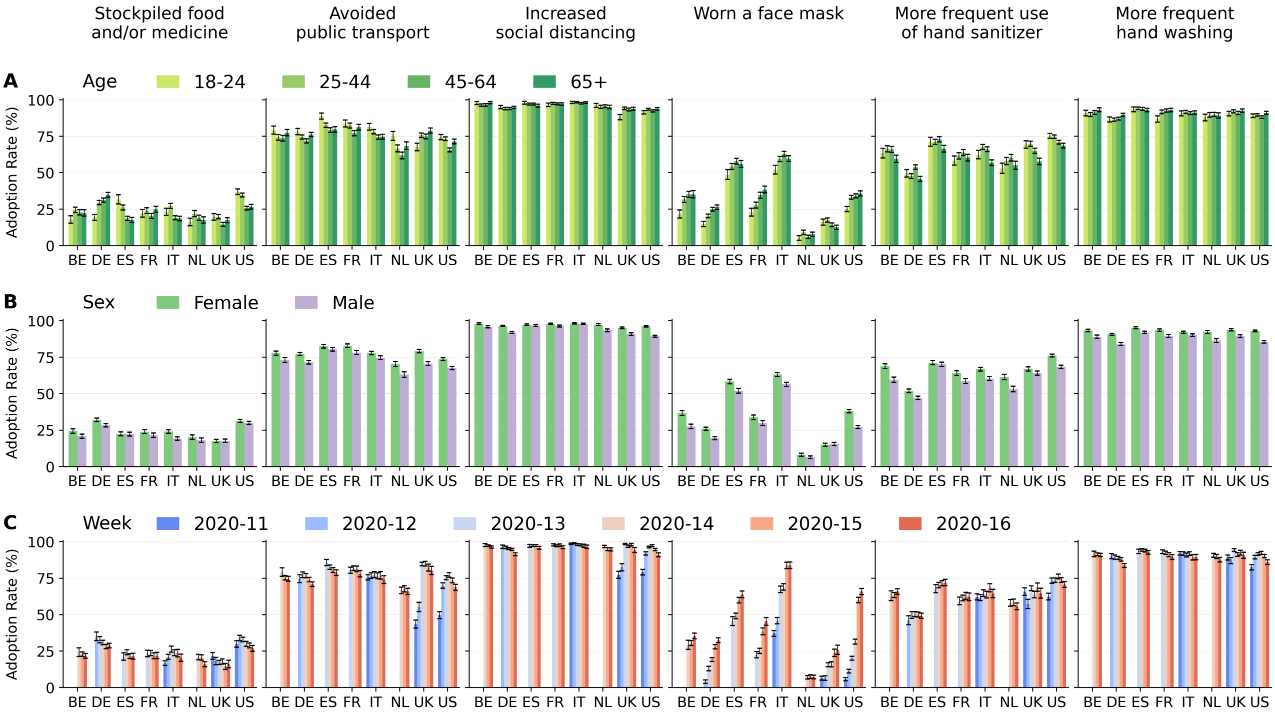

Fig. S11. Adoption of preventive behaviours. Adoption rate of behaviours broken down by age (**A**), sex (**B**), and calendar week (**C**). The adoption rate is defined as the weighted proportion of individuals who adopted a specific behaviour. Bar charts show mean values as bars and bootstrapped 95%CI as errors. Weighted sample.

Table S4. Correspondence of micro- and macro-regions for all countries included in the analysis.

Country	Macro-region	Micro-region
Belgium	Brussels	Brussels
	Flanders	Antwerpen, Limburg, Oost-Vlaanderen, Vlaams-Brabant, West-Vlaanderen
	Wallonia	Wallon Brabant, Hainaut, Liège, Luxembourg, Namur
France	Île de France	Île de France
	North East	Alsace, Bourgogne, Champagne-Ardenne, Franche-Comte, Lorraine, Nord-Pas-de-Calais, Picardie
	South West	Aquitaine - Limousin - Poitou-Charentes, Languedoc-Roussillon - Midi-Pyrénées
	South East	Auvergne - Rhône-Alpes, Corse, Provence-Alpes-Côte d'Azur
	West	Bretagne, Centre - Val de Loire, Normandie, Pays-de-la-Loire
Germany	Norrdeutschland	Bremen, Hamburg, Mecklenburg-Vorpommern, Niedersachsen, Schleswig-Holstein
	Ostdeutschland	Berlin, Brandenburg, Sachsen, Sachsen-Anhalt, Thüringen
	Süddeutschland	Baden-Württemberg, Bayern
	Westdeutschland	Hessen, Nordrhein-Westfalen, Rheinland-Pfalz, Saarland
Italy	Central	Lazio, Marche, Toscana, Umbria
	Insular	Sardegna, Sicily
	Northeast	Emilia-Romagna, Friuli-Venezia Giulia, Trentino-Alto Adige, Veneto
	Northwest	Liguria, Lombardia, Piemonte, Valle d'Aosta
	South	Abruzzo, Basilicata, Calabria, Campania, Molise, Apulia
Netherlands	East	Flevoland, Gelderland, Overijssel
	North	Drenthe, Friesland,Groningen
	South	Limburg, Noord-Brabant
	West	Noord-Holland, Utrecht, Zuid-Holland, Zeeland
Spain	Center	Castilla y León, Castilla-la Mancha, Extremadura
	East	Cataluña, Comunidad Valenciana, Illes Balears
	Island	Canarias
	Madrid	Comunidad de Madrid
	Northwest	Cantabria, Galicia, Principado de Asturias
	North East	Aragón, Comunidad Foral de Navarra, La Rioja, País Vasco
	South	Andalucía, Región de Murcia
United Kingdom	England	East Midlands, East of England, North East, North West, South East, South West, West Midlands, Yorkshire and The Humber
	London	London
	Northern Ireland	Northern Ireland
	Scotland	Scotland
	Wales	Wales
United States	Midwest	Illinois, Indiana, Iowa, Kansas, Michigan, Minnesota, Missouri, Nebraska, North Dakota, Ohio, South Dakota, Wisconsin
	Northeast	Connecticut, Maine, Massachusetts, New Hampshire, New Jersey, New York, Pennsylvania, Rhode Island, Vermont
	South	Alabama, Arkansas, Delaware, Florida, Georgia, Kentucky, Louisiana, Maryland, Mississippi, North Carolina, Oklahoma, South Carolina, Tennessee, Texas, Virginia, District of Columbia, West Virginia
	West	Alaska, Arizona, California, Colorado, Hawaii, Idaho, Montana, Nevada, New Mexico, Oregon, Utah, Washington, Wyoming

References

[1] S. Pötzschke and M. Braun, "Migrant sampling using Facebook advertisements: A case study of Polish migrants in four European countries," *Social Science Computer Review*, vol. 35, no. 5, pp. 633–653, 2017.

[2] D. A. Dillman, J. D. Smyth, and L. M. Christian, *Internet, Phone, Mail, and Mixed-Mode Surveys: The Tailored Design Method.* John Wiley & Sons, 2014.

[3] European Union, "Eurostat regional yearbook, 2019." `https://ec.europa.eu/eurostat/web/products-statistical-books/-/KS-HA-19-001`, 2019. (Accessed on March 20, 2020).

[4] U.S. Census Bureau, Population Division, "Annual Estimates of the Resident Population by Sex, Age, Race, and Hispanic Origin for the United States and States: April 1, 2010 to July 1, 2018," 2019.

[5] European Social Survey, "Round 9: European Social Survey Round 9 Data (2018)." `https://www.europeansocialsurvey.org/download.html?file=ESS9e01_2&y=2018`. (Accessed on March 20, 2020).

[6] U.S. Census Bureau, "American Community Survey 1-Year Estimates, 2018." `https://data.census.gov/cedsci/`. (Accessed on March 20, 2020).
