# Supplementary material for: Behaviours and attitudes in response to the COVID-19 pandemic: insights from a cross-national Facebook survey
Source: EPJ Data Sci. 2021 Apr 14;10(1):17. doi: 10.1140/epjds/s13688-021-00270-1 (PMC8050509; doi:10.1140/epjds/s13688-021-00270-1)
Supplement: Supplementary file 2 — The English version of the questionnaire used in the study. (PDF 411 kB) [file 13688_2021_270_MOESM2_ESM.pdf]

S1. Intro

Health Behaviour Survey United States

Thank you for your interest in our survey. This is an academic study led by researchers from the Max Planck Institute for Demographic Research. Our goal is to understand health behaviors and help improve the health and well-being of people across countries at a time of increasing uncertainty. Your participation is crucial for our study.

The survey is directed at all people who are 18 years old or older, and it will take approximately 10-12 minutes to complete. Your participation is completely voluntary, you can stop participating at any time. In case you are not comfortable answering a particular question, you have the option to select "Prefer not to answer". Participants' data will be treated anonymously, and we will not ask for identifying information. If you wish to provide your email address, it will not be linked to your survey data. You can download our data protection policy here.

If you have any questions about this research study, please contact Dr. André Grow and Dr. Daniela Perrotta at the Max Planck Institute for Demographic Research via healthsurvey@demogr.mpg.de.

Tick the box below and press 'Next' to continue.

□ I am willing to participate in this survey, I am at least 18 years old, and I have read the data protection policy.

S2. About you

First of all, we would like to ask you some questions about you, your background and your daily life.

Q1. How old are you?
[NUMERICAL FIELD]

Q2. What is your sex?
1. Male
2. Female
3. Prefer not to answer

Q3. What is your country of birth?
[COUNTRY LIST]

Q4. In which country do you currently live?
[COUNTRY LIST]

Q5. What is your place of residence?
[REGION LIST]

FILTER: if the country selected in Q5 coincides with the country of the survey.
Q6. What is the postcode of your place of residence?
[OPEN TEXT FIELD]

Q7. What is the postcode of the place where you spend the majority of a typical weekday (e.g. your workplace, school, or home)?
[OPEN TEXT FIELD]

Q8. Which of these descriptions applies to what you have been doing for the last 7 days? Check all that apply.
1. In paid work (or away temporarily) (employee, self-employed, working for your family business)
2. In education (not paid for by employer) even if on vacation
3. Unemployed and actively looking for a job
4. Unemployed, wanting a job but not actively looking for a job
5. Permanently sick or disabled
6. Retired
7. In community or military service
8. Doing housework, looking after children or other persons

9. Other: [OPEN TEXT FIELD]
10. Don’t know
11. Prefer not to answer

Q9. What is the highest educational level that you have attained?

1. No schooling completed
2. Nursery school
3. Kindergarten
4. 12th grade - NO DIPLOMA
5. Regular high school diploma
6. GED or alternative credential
7. Some college credit, but less than 1 year of college credit
8. 1 or more years of college credit, no degree
9. Associate’s degree (for example: AA, AS)
10. Bachelor’s degree (for example: BA, BS)
11. Master’s degree (for example: MA, MS, MEng, MEd, MSW, MBA)
12. Professional degree beyond a bachelor’s degree (for example: MD, DDS, DVM, LLB, JD)
13. Doctorate degree (for example: PhD, EdD)
14. Prefer not to answer

Q10. Including yourself, how many people – including children – live regularly at your home?

1. Number of people: [NUMERICAL FIELD]
2. Prefer not to answer

FILTER: if option 1 was selected in Q10.

Q11. How many people living in your house belong to each of the following age groups?

0-15 years	[NUMERICAL FIELD]
16-64 years	[NUMERICAL FIELD]
65+ years	[NUMERICAL FIELD]

S3. COVID-19: The outbreak of a novel coronavirus

Following the first reports of cases of acute respiratory syndrome in the Chinese Wuhan municipality at the end of December 2019, a novel coronavirus has been identified by the Chinese authorities as the cause of the outbreak. The virus has rapidly spread, affecting other parts of China, as well as other countries. The disease caused by this novel coronavirus has been called COVID-19.

Q12. How much, if at all, have you seen, read or heard about the coronavirus outbreak?

1. Nothing at all
2. Not very much
3. A fair amount
4. A great deal
5. Prefer not to answer

FILTER: if option 2,3,4 was selected in Q12.

S4. COVID-19 from your point of view

We value your opinion on the coronavirus outbreak that has been recently affecting people's lives. We would like to ask you now some questions related to your behaviours in response to the epidemic.

Q13. What level of threat do you think the coronavirus poses to each of the following?

	1 - Very low threat	2 - Low threat	3 - Moderate threat	4 - High threat	5 - Very high threat	Don't know	Prefer not to answer
The world							
Your country							
Your local community							
You personally							
Your family							

Q14. Based on what you have seen, read or heard, how confident are you the following are prepared and can effectively deal with the coronavirus?

	1 – Not confident at all	2 – Little confident	3 – Somewhat confident	4 – Very confident	Don't know	Prefer not to answer
Your national government						
Your local government						
The World Health Organization						
Healthcare services in your country						
Hospitals in your local area						
Doctors & healthcare professionals in your community						

Airports in your country						
Airlines in your country						
Your friends and neighbours						

Q15. How much confidence, if any, do you have in the accuracy of these sources of information on the coronavirus?

	1 - Nothing at all	2 - Not very much	3 - A fair amount	4 - A great deal	Don't know	Prefer not to answer
Social media networks						
Friends, family and colleagues						
Government health officials						
Radio						
Magazines						
Newspapers						
Television						
The Internet						
Medical professionals such as doctors and nurses						

Notes: Item order randomized

Q16. Do you agree or disagree with the following:

	1 - Strongly disagree	2 - Somewhat disagree	3 - Somewhat agree	4 - Strongly agree	Don't know	Prefer not to answer
I am confident pharmaceutical companies will soon develop a vaccine or treatment for the coronavirus.						
The coronavirus outbreak has been contained and will soon						

be over.						
The media has exaggerated the extent of the coronavirus outbreak.						
The recommended preventive measures are effective to reduce population contagion						

Notes: Item order randomized

Q17. On March 9, 2020, Italy was the first European country to declare a nationwide lockdown as a control measure to curb the coronavirus epidemic in the country. Do you agree or disagree with the following?

	1 - Strongly disagree	2 - Somewhat disagree	3 - Somewhat agree	4 - Strongly agree	Don't know	Prefer not to answer
The USA is at risk of being locked down soon to contain the spread of coronavirus						
The USA is better equipped to deal with the coronavirus outbreak than Italy						

Q18. Which of the following actions, if any, have you already taken to protect yourself from the coronavirus? Check all that apply.
We do not seek to suggest that any of the above actions do, or do not, help prevent the spread of the virus or the infection. We only seek to understand which actions people actually engage in. For more information on the recommended preventive measures, please refer to the official guidance of your national health representatives.

1. Avoided purchasing products from Asia (e.g. food, goods)
2. Avoided purchasing products from Italy (e.g. food, goods)
3. Avoided purchasing products from Iran (e.g. food, goods)
4. Avoided social activities (e.g. meeting friends)
5. Avoided eating in Asian restaurants
6. Avoided eating in Italian restaurants
7. Avoided eating in Iranian restaurants

8. Avoided travelling by public transportation (e.g. bus, tram, subway, train)
9. Stockpiling of medicines
10. Stockpiling of food
11. Avoided crowded places (e.g. restaurant, cinema, gym, playground, stadium)
12. Avoided travelling by taxi
13. Bought a face mask
14. Worn a face mask
15. Washed hands more often
16. Used sanitizing hand gel more often
17. Avoided shaking hands
18. Avoided contacts with people of Asian origin or appearance
19. Avoided contacts with people of Italian origin or appearance
20. Avoided contacts with people of Iranian origin or appearance
21. Other: [OPEN TEXT]
22. None
23. Prefer not to answer

Notes: Items 2, 6, and 19 not asked in Italy

Q19. Have you changed or cancelled any of your scheduled trips in 2020 to protect yourself from the coronavirus?

1. Yes
2. No
3. Not applicable (no trips planned)
4. Prefer not to answer

FILTER: if option 1 was selected in question Q19.

Q20. What was the trip's destination that you have changed or cancelled?

[COUNTRY LIST]

FILTER: if option 1 was selected in question Q19.

Q21. For when was the trip that you changed or cancelled scheduled?

[MONTH LIST]

FILTER: if option 1 or 2 was selected in question Q19.

Q22. Do you have other trips planned in 2020?

1. Yes
2. No
3. Prefer not to answer

FILTER: if option 1 was selected in question Q22.

Q23. What is the destination country of your next trip this year?

[COUNTRY LIST]

FILTER: if option 1 was selected in question Q22.
Q24. For when is your next trip this year scheduled?
[MONTH LIST]

Q25. Due to the coronavirus outbreak, have you personally experienced any of the following disruptions to your daily life? Check all that apply.

1. School closure (either affecting you as a student or as a parent)
2. Workplace closure
3. Working remotely (e.g. smart working)
4. Quarantine
5. Other: [OPEN TEXT]
6. None
7. Prefer not to answer

Q26. Have you been tested for coronavirus?

1. Yes
2. No
3. Prefer not to answer

FILTER: if option 1 was selected in question Q26.
Q27. Did you test positive for coronavirus?

1. Yes
2. No
3. Don't know (test results not available yet)
4. Prefer not to answer

S5. About your health

We would like to ask you some questions related to your health and health behaviors.

Q28. Do you suffer from any chronic diseases (e.g. diabetes, cardiovascular or respiratory diseases, cancer, etc.)?

1. Yes
2. No
3. Don't know
4. Prefer not to answer

Q29. Have you received the flu vaccine in the last 6 months?

1. Yes
2. No
3. Don't know
4. Prefer not to answer

Q30. What level of threat do you think the flu poses to each of the following?

	1 - Very low threat	2 - Low threat	3 - Moderate threat	4 - High threat	5 - Very high threat	Don't know	Prefer not to answer
The world							
Your country							
Your local community							
You personally							
Your family							

FILTER: if option 2 was selected in Q2 (i.e. female).

Q31. Are you currently pregnant?

1. Yes
2. No
3. Don't know
4. Prefer not to answer

Q32. Have you experienced any of the following symptoms in the last 7 days? Check all that apply.

1. Runny or blocked nose
2. Stomach ache
3. Nausea

4. Headache
5. Tiredness
6. Cough
7. Malaise
8. Muscle/joint pain
9. Sore throat
10. Difficult breathing
11. Fever
12. Sneezing
13. Others: [OPEN TEXT]
14. No symptoms
15. Prefer not to answer

FILTER: if at least one symptom was selected in question Q32.

Q33. Because of these symptoms, have you used any of the following services? Check all that apply.

1. Emergency hotline (e.g. 112, 999, 911)
2. Internet (e.g. Google, Wikipedia)
3. General Practitioner
4. Hospital, emergency room
5. Pharmacy
6. Others: [OPEN TEXT]
7. None
8. Prefer not to answer

S6. About your social interactions

As a last request, we would like you to record the number of persons you had contact with yesterday in different locations (at home, at school/college, at work, other locations), from when you woke up to when you went to sleep.

- A contact is defined as:
    - EITHER a two-way conversation with three or more words in the physical presence of another person,
    - OR physical skin-to-skin contact (for example a handshake, hug, kiss or contact sports).
- Think about every person that you contacted during the day, regardless of whether the contact was long or short, and whether you knew the person or not.
- Contacts made exclusively by telephone or mobile phone should NOT be recorded.
- If you contacted the same person in different locations in the course of the day, only count him/her once, and assign him/her to the location where you two had the contact of longer duration.
    - Example: Anne runs a family business together with her husband Tom. Anne and Tom saw each other yesterday both at home and at work. Since they spent more time together at home than at work, Anne will count the contact with Tom as a home contact, and not a work contact.
- If you had no contacts in a given location, please enter 0.

How many persons did you have contacts with yesterday ...

Q34. …at your home?

1. Number of people: [NUMERICAL FIELD]
2. Prefer not to answer

Q35. … at school/college?

1. Number of people: [NUMERICAL FIELD]
2. Not applicable (e.g. you do not attend any school)
3. Prefer not to answer

Q36. … at work? (at your workplace or throughout the course of your daily job activity, such as meeting customers or clients)

1. Number of people: [NUMERICAL FIELD]
2. Not applicable (e.g. you do not work)
3. Prefer not to answer

Q37. … in other settings? (e.g. during transportation and commuting, social and leisure activities, religious services, shopping, or other activities)

1. Number of people: [NUMERICAL FIELD]
2. Prefer not to answer

FILTER: If a number of contacts greater than 0 was inserted in question Q34.

Q38. You indicated above that yesterday you had contacts with some people at home. Did any of them show any of the following symptoms: fever, cough, tiredness, difficult breathing, or muscle pain?

1. Yes
2. No
3. Don't know
4. Prefer not to answer

S7. Keep in touch!

To assess changes in health and health behaviours in our populations over time, we may ask you to participate in this or a similar survey again in the future. Your participation is crucial for our research! If you are willing to be contacted again, please check the box below. You can request the deletion of your email address at any time. For more information please read our data protection policy here.

Q39. Do you agree to provide your email address?

1. Yes, I agree to provide my email address
2. No, I will not provide my email address

FILTER: if option 1 was selected in question Q39.

Q40. Please enter your email address below.

[OPEN TEXT FIELD]
